# Supplementary material for: Inverse Impact of Cancer Drugs on Circular and Linear RNAs in Breast Cancer Cell Lines
Source: Noncoding RNA. 2023 May 19;9(3):32. doi: 10.3390/ncrna9030032 (PMC10204552; doi:10.3390/ncrna9030032)
Supplement: Supplementary file 1 [file ncrna-09-00032-s001.zip › ncrna-2201428-supplementary/Table S1 - MCF7.pdf]

Table S1. Mutations with documented activity relevant to cancer in MCF-7 cells. Selected mutations by Census tiers 1 from COSMIC (https://cancer.sanger.ac.uk/cosmic).

Gene symbol	Gene Name	AA mutation	CDS mutation	Type
**ATP2B3**	ATPase plasma membrane Ca2+ transporting 3	p.V882E	c.2645T>A	Substitution - Missense
**EP300**	E1A binding protein p300	p.R1356*	c.4066C>T	Substitution - Nonsense
**ERBB4**	Erb-b2 receptor tyrosine kinase 4	p.Y1242C	c.3725A>G	Substitution - Missense
**FUS**	FUS RNA binding protein	p.D502del	c.1503_1505del	Deletion - In frame
**GATA3**	GATA binding protein 3	p.D336Gfs*17	c.1006dup	Insertion - Frameshift
**KMT2C**	Lysine methyltransferase 2C	p.I3590L	c.10768A>C	Substitution - Missense
**LATS1**	Large tumor suppressor kinase 1	p.D1043N	c.3127G>A	Substitution - Missense
**MAP3K13**	Mitogen-activated protein kinase kinase kinase 13	p.D380N	c.1138G>A	Substitution - Missense
**MYH9**	Myosin heavy chain 9	p.K682_L687del	c.2044_2061del	Deletion - In frame
**PIK3CA**	Phosphatidylinositol-4,5-bisphosphate 3-kinase catalytic subunit alpha	p.E545K	c.1633G>A	Substitution - Missense
