# Supplementary material for: Inverse Impact of Cancer Drugs on Circular and Linear RNAs in Breast Cancer Cell Lines
Source: Noncoding RNA. 2023 May 19;9(3):32. doi: 10.3390/ncrna9030032 (PMC10204552; doi:10.3390/ncrna9030032)
Supplement: Supplementary file 1 [file ncrna-09-00032-s001.zip › ncrna-2201428-supplementary/Table S2 - MDA-MB-231.pdf]

Table S2. Mutations with documented activity relevant to cancer in MDA-MB-231 cells. Selected mutations by Census tiers 1 from COSMIC (https://cancer.sanger.ac.uk/cosmic).

Gene symbol	Gene Name	AA mutation	CDS mutation	Type
BRAF	B-Raf proto-oncogene, serine/threonine kinase	p.G464V	c.1391G>T	Substitution - Missense
CD79A	CD79a molecule	p.C106Y	c.317G>A	Substitution - Missense
CRTC3	CREB regulated transcription coactivator 3	p.P578A	c.1732C>G	Substitution - Missense
NF2	NF2, moesin-ezrin-radixin like (MERLIN) tumor suppressor	p.E231*	c.691G>T	Substitution - Nonsense
PDGFRA	Platelet derived growth factor receptor alpha	p.Y172F	c.515A>T	Substitution - Missense
TP53	Tumor protein p53	p.R280K	c.839G>A	Substitution - Missense
