# Supplementary material for: Inverse Impact of Cancer Drugs on Circular and Linear RNAs in Breast Cancer Cell Lines
Source: Noncoding RNA. 2023 May 19;9(3):32. doi: 10.3390/ncrna9030032 (PMC10204552; doi:10.3390/ncrna9030032)
Supplement: Supplementary file 1 [file ncrna-09-00032-s001.zip › ncrna-2201428-supplementary/Table S3 - primers.pdf]

Table S3. Primers used to amplify circular and linear RNAs.

Type of RNA	Gene name	Forward primer	Reverse primer	Melting temperature	Reference
Housekeeping	GAPDH	5′-CTATAAATTGAGCCCGCAGCC-3′	5′-CCCAATACGACCAAATCCGT-3′	60°C	Gorji-Bahri et al. 2021 [50]
circular	000911	5'-AAAAGCAAGCAGTGCCCATA-3'	5'-GCTCGAATCAGGTCCACCA-3'	60°C	Wang et al. 2018 [18]
circular	VRK1	5'-GAACCTGGTGTTGAAGATACGG-3'	5'-AATCCTACTTTCCATTCCTTTTTTG-3'	60°C	Yan et al. 2017 [17]
circular	BCL11B	5'-ACGAAAGGCATCTGTCCCAA-3'	5'-TTGTGCTCTATAAAAACCAGGATGT-3'	60°C	Yan et al. 2017 [17]
circular	GFRA1	5'-CCTCCGGGTTAAGAACAAGC-3'	5'-CTGGCTGGCAGTTGGTAAAA-3'	60°C	He et al. 2017 [21]
circular	IGF1R	5'-AGCCGATGTGTGAGAAGACC-3'	5'-CAGCTGCTGATAGTCGTTGC-3'	60°C	Coscujuela Tarrero et al. 2018 [14]
circular	ESR1	5'-CAACCAGTGCACCATTGATA-3'	5'-TCCTTGGCAGATTCCATAG-3'	60°C	Coscujuela Tarrero et al. 2018 [14]
circular	HIPK3	5'-GGCAGCCTTACAGGGTTAA-3'	5'-GGGTAGACCAAGACTTGTGAG-3'	60°C	Coscujuela Tarrero et al. 2018 [14]
circular	NCOA3	5'-GCCTGGCTTTGAAGATATAATC-3'	5'-TCAGCTCAGCCAATTCTTC-3'	60°C	Coscujuela Tarrero et al. 2018 [14]
circular	MAN1A2	5'-TCTGTGTTTGAAGTCAACATTCG-3'	5'-GCTTCTTCCAAGGCCTTCTC-3'	60°C	Coscujuela Tarrero et al. 2018 [14]
circular	AKT2	5'-GCCCCTGATCAGACTCTACC-3'	5'-CAGGGCACAGTCTCTCGTC-3'	62°C	Vo et al. 2019 [19]
circular	AKT3	5'-GCAAAGGATGAAGTGGCACA-3'	5'-TGATGACTCAGCCTCGCC-3'	60 °C	Vo et al. 2019 [19]
circular	PIK3CB	5'-CTGGTTTGGATCTTCGGTTGA-3'	5'-TCCCGGTAAGCACTCTGTTT-3'	60°C	Vo et al. 2019 [19]
linear	SNX27	5'-GAGCAGGCGAGAAGGAATTG-3'	5'-GCTTAGAACACAGCTGCCTC-3'	60°C	Lee 2016 et al. [51]
linear	VRK1	5'-CTACCAACGAGCTGCAAAACC-3'	5'-TCACTCCCAAAGCGATCCATTA-3'	60°C	Li 2017 et al. [52]
linear	BCL11B	5'-TGCCAGTGTCAGTTGTCAGG-3'	5'-CCAGGTAGATGCGGAAGC-3'	60°C	Permatasari 2017 *et al.* [53]
linear	GFRA1	5'-CCAAAGGGAACAACTGCCTG-3'	5'-CGGTTGCAGACATCGTTGGA-3'	60°C	He et al. 2017 [21]

linear	IGF1R	5'-ACGAGTGGAGAAATCTGCGG-3'	5'-ATGTGGAGGTAGCCCTCGAT-3'	60°C	Coscujuela Tarrero et al. 2018 [14]
linear	ESR1	5'-GACAGGGAGCTGGTTCACAT-3'	5'-CCAGACGAGACCAATCATCA-3'	60°C	Coscujuela Tarrero et al. 2018 [14]
linear	HIPK3	5'-TGGAGACTGGGGGAAGATGA-3'	5'-CACACTAACTGGCTGAGGGG-3'	60°C	Liu et al. 2018 [54]
linear	NCOA3	5'-ACTTGCTGGATGGTGGACT-3'	5'-ACATGGCAATTTGCGTTTT-3'	60°C	Coscujuela Tarrero et al. 2018 [14]
linear	MAN1A2	5'-CCGGTAAAGGGGCTAAAAAC-3'	5'-GCTTCTTCCAAGGCCTTCTC-3'	60°C	Coscujuela Tarrero et al. 2018 [14]
linear	AKT2	5'-ACAAGGAAAGGGAACCAGCG-3'	5'-GGTACGCTGTCACCTAGCTC-3'	60 °C	Vo *et al*. 2019 [19]
linear	AKT3	5'-GCAAAGGATGAAGTGGCACA-3'	5'-ACCCGCTCTCTCGACAAATG-3'	64.5°C	Vo *et al*. 2019 [19]
linear	PIK3CB	5'-CAGCTGAGATTGCAAGCAGTG-3'	5'-ATCTCTCGGCAGTCTTGTCG-3'	64.5°C	Vo *et al*. 2019 [19]
